# High-*Q* Fano Resonance in Terahertz Frequency Based on an Asymmetric Metamaterial Resonator

**DOI:** 10.1186/s11671-018-2677-0

**Published:** 2018-09-21

**Authors:** Qin Xie, Guang-Xi Dong, Ben-Xin Wang, Wei-Qing Huang

**Affiliations:** 1 School of Science, Jiangnan University, Wuxi, 214122 China; 2 School of Physics and Electronics, Hunan University, Changsha, 410082 China

**Keywords:** Metamaterial, Fano, Terahertz, Sensing

## Abstract

We propose a planar metamaterial formed by four-strip metallic resonators, which can achieve high-*Q* Fano resonance in terahertz regime. This terahertz planar metamaterial supports a sharp Fano resonance at 0.81 THz with 25% transmission. The resonance bandwidth of the dip is 0.014 THz with the *Q*-factor of 58. The interference between the bright mode and dark mode leads to the Fano line shape. This sharp Fano profile is explained by the electromagnetic theory of Fano resonance. Moreover, multiple Fano resonances can be realized by adding more strips into the original structure. As an example, two Fano dips with *Q*-factors of 61 and 65 can be achieved via a five-strip structure.

**Electronic supplementary material:**

The online version of this article (10.1186/s11671-018-2677-0) contains supplementary material, which is available to authorized users.

## Background

Metamaterial is a kind of artificial material exhibiting exotic properties, such as negative refractive index [1] and ultra-high refractive index [2], which cannot be realized by natural materials in most situations. Such artificial material is composed of a large amount of periodic metallic units, and its characteristics (e.g., permittivity and permeability) can be easily controlled by changing the geometric parameters of the units [3]. As a result, the study of metamaterials has attracted wide attention in recent years. A great many novel applications have emerged in this domain, including perfect absorption [4, 5], metamaterial sensors [6–9], cloaking [10], Fano effects [11], etc.

The line shape of Fano resonance is quite different from symmetric Lorentzian profile. It is asymmetric and sharp with a relatively high *Q*-factor. Since Fano theoretically revealed the quantum mechanism of Fano resonance [12], it has become a hot topic. To illustrate the origin of Fano resonance, several theories have been set up, including Fano’s quantum mechanical analysis [12], classical oscillator model [13], coupled-mode theory [14], and electromagnetic theory of Fano resonance [15, 16]. According to the electromagnetic theory of Fano resonance proposed by Gallinet and Martin [16], the distinctive Fano profile is attributed to the coupling between a nonradiative mode and a radiative mode which can also be seen as a continuum.

In terahertz regime, the sharp Fano resonance can be achieved by introducing a weak asymmetry in metamaterials [17–20], which may lead to the appearance of an underlying dark mode [21]. Besides, graphene materials can also be utilized to generate and even modulate the Fano resonance [22, 23]. Compared with a majority of EIT (electromagnetically induced transparency) [24, 25] and PIT (plasmon-induced transparency) [26, 27], Fano line shape is much sharper and narrower. The *Q*-factor of Fano [17, 28] profile is approximately ten times larger than that of Lorentzian line shape [29–31] in many situations. This property makes Fano resonance a promising choice to realize sensitive detecting [8]. However, the *Q*-factor of a lot of metamaterials is not high enough [17, 32, 33], which limits their applications in terms of sensing. In order to widely and efficiently apply Fano resonance into sensing, it is a necessary task to greatly improve the *Q*-factor of a metasurface.

Recently, some metamaterial structures have been designed to realize high-*Q* Fano resonance. For instance, Ding et al. proposed a bilayer metamaterial which consists of two sets of asymmetric split-rings with different geometric parameters. It can support three Fano resonances whose *Q*-factors are respectively 33, 42, and 25 [19]. A symmetric dimer structure composed of identical spit-ring resonators on each layer was also presented to improve its *Q*-factor [34]. However, these stacked structures are suffering from technical challenges in manufacturing. High-*Q* resonance with simple structure design still remains to be a hot issue.

In this paper, we demonstrate a coplanar metamaterial structure consisted of four metallic strips. In each unit cell, three parallel strips are arranged perpendicular to the fourth one. This structure can support a high-*Q* Fano resonance (*Q*-value is about 58) at 0.81 THz with 25% transmission. This sharp line shape originates from the interaction between bright (radiative) mode and dark (nonradiative) mode. For further discussion, the electromagnetic theory of Fano resonance is employed [15, 16]. The properties of Fano resonance can be changed via controlling of geometric parameters. The sensing performance of the device is discussed. Moreover, by adding more strips into the originally designed structure, multiple Fano resonances can be realized.

## Methods/Experimental

**Fig. 1:**
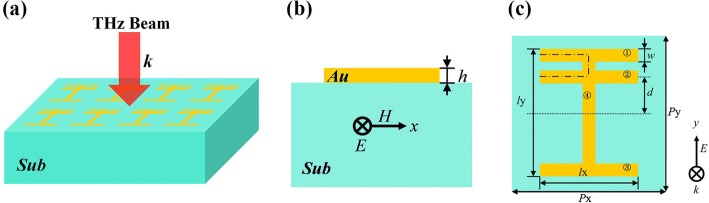
Three-dimensional diagram of the proposed metamaterial (**a**). Side view (**b**) and top view (**c**) of the asymmetric metamaterial resonator; the equivalent length *l* is marked by dot-dash line

A large amount of researches indicate that breaching the symmetry of a structure may induce an asymmetric Fano line shape [17, 18, 35–37]. Based on this concept, we design this four-strip metamaterial displayed in Fig. 1, where strip 2 is set to realize a symmetry breaking. Figure 1a shows the three-dimensional diagram of the proposed metamaterial. Figure 1b, c respectively shows the side view and top view of the structure unit. The metallic four-strip resonators are placed on the top of an ideal dielectric substrate whose real part of refractive index is 1.5 and imaginary part is 0. In reality, this dielectric material is corresponding to silica. That is to say, the substrate is lossless in terahertz region. We choose Au with conductivity *σ* = 4.09 × 10^7^ S/m to form the metallic planar resonator whose thickness is 0.2 μm. The repeat period is *P*_x_ = *P*_y_ = 180 μm. Three parallel strips (1, 2, and 3) have the same size. Their length is *l*_x_ = 120 μm and width is *w* = 20 μm. Strip 4 is perpendicular to the other strips (1, 2, and 3). Its length is *l*_y_ = 150 μm and width is *w* = 20 μm. The distance between the axis of strip 2 and the central point of the structure is *d* = 30 μm. Finite-difference time-domain method is used to simulate this planar metamaterial. In order to save simulation time and computing memory, we choose the mesh sizes of Δ*x* = Δ*y* = 1 μm and Δ*z* = 0.02 μm. We find the simulation results are quite accurate in this case. Even if smaller mesh sizes are applied, the simulation results are nearly unchanged. The simulating boundary conditions along *x*-axis and *y*-axis are set as periodic, and the condition along *z*-axis is set as perfectly matched layers. Figure 1a shows that the whole structure is illuminated by a beam of normally incident THz wave. As seen in Fig. 1b, c, the electric vector ***E*** and magnetic vector ***H*** of the incident THz beam are *y*-axis polarized and *x*-axis polarized, respectively.

## Results and Discussion

**Fig. 2:**
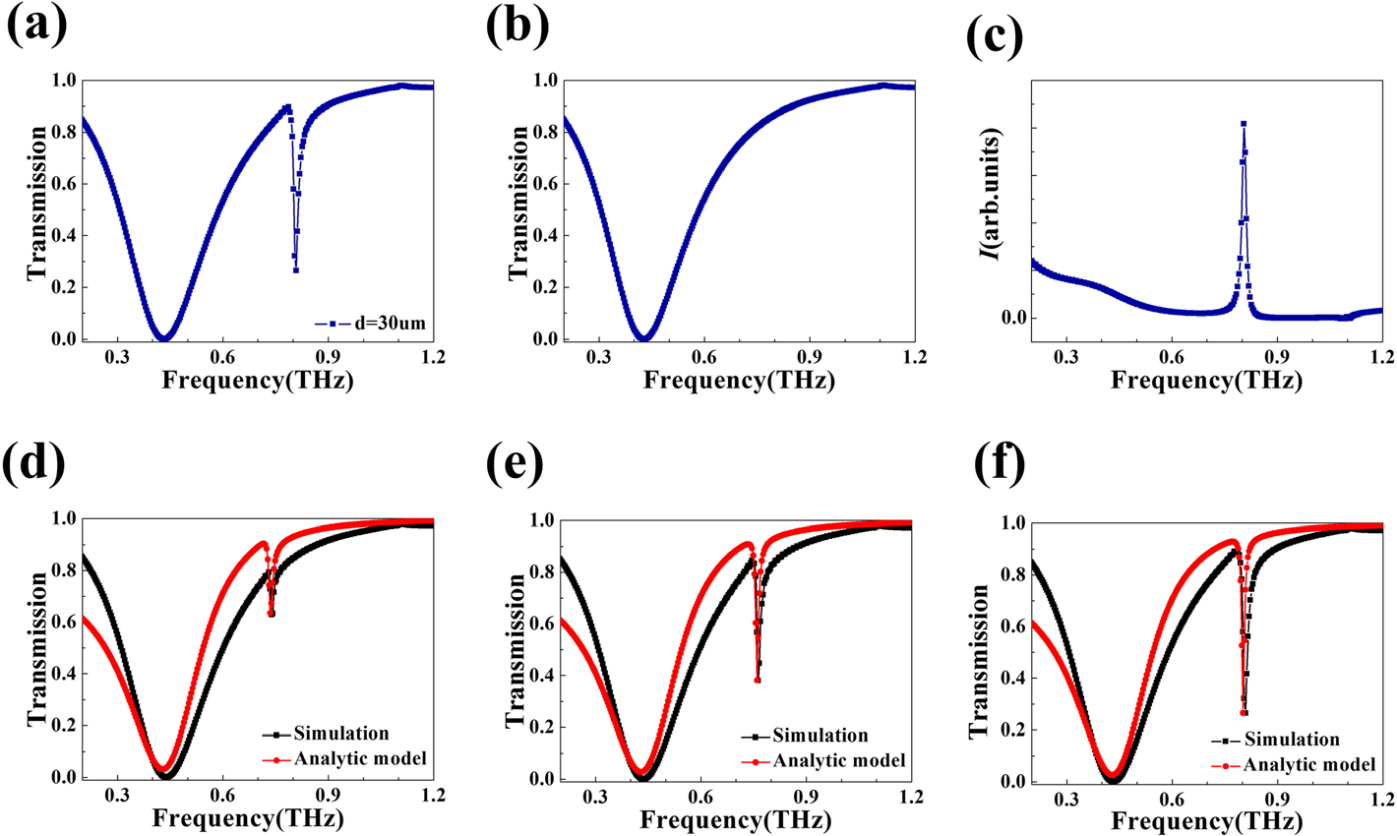
**a** Transmittance curve of the designed metasurface given by numerical simulation. **b** Transmission spectrum of the bright mode. **c** Field intensity of the proposed four-strip nanostructure illuminated by a dipole source. **d**, **e**, **f** Simulated (red curve) and theoretical (black curve) transmission spectrum of the designed structure with *d* = 10 μm, *d* = 20 μm, and *d* = 30 μm, respectively

The transmission spectrum of the proposed metasurface is shown in Fig. 2a. There are two transmission dips at the frequencies of 0.430 THz and 0.809 THz with the transmission rates of 0.10% and 26.45%, respectively. In order to make the following explanation more concise, we use *R*_s_R_s_ and *R*_d_ to mark these two resonant modes, *R*_s_ for the mode resonant at 0.430 THz and *R*_d_ for the resonant mode at a higher frequency. The optical transmission rate of *R*_s_ shows a symmetric Lorentz profile with a relatively wide bandwidth of 0.256 THz. By comparison, *R*_d_ exhibits an asymmetric Fano line shape which is much sharper with a bandwidth of 0.014 THz. *Q*-factor is an important criterion to judge the line shape. It can be obtained through dividing the central frequency by bandwidth. In fact, the *Q*-factor of *R*_d_ can reach 58, 30 times more than the *Q*-value of *R*_s_, which contributes to underlying applications in many fields. The existence of the asymmetric Fano profile roots in the interaction between a dark mode and a bright mode, i.e., the interaction between a nonradiative state and a continuum, generates from a radiative state [16, 38, 39]. In the rest of the paper, detailed mechanism of the Fano line shape will be discussed and theoretical transmission spectra will be analyzed. Although the transmission at 0.809 THz is 26.45% in the proposed metasurface, it can be reduced further. According to [40, 41], the utilization of lossy dielectric materials may reduce transmission. In our simulations, the substrate material we choose is an ideal material with a real refractive index of 1.5 that has no loss in terahertz region. A feasible method to reduce transmission is using a lossy material with a complex refractive index to form the substrate rather than this ideal lossless material.

**Fig. 3:**
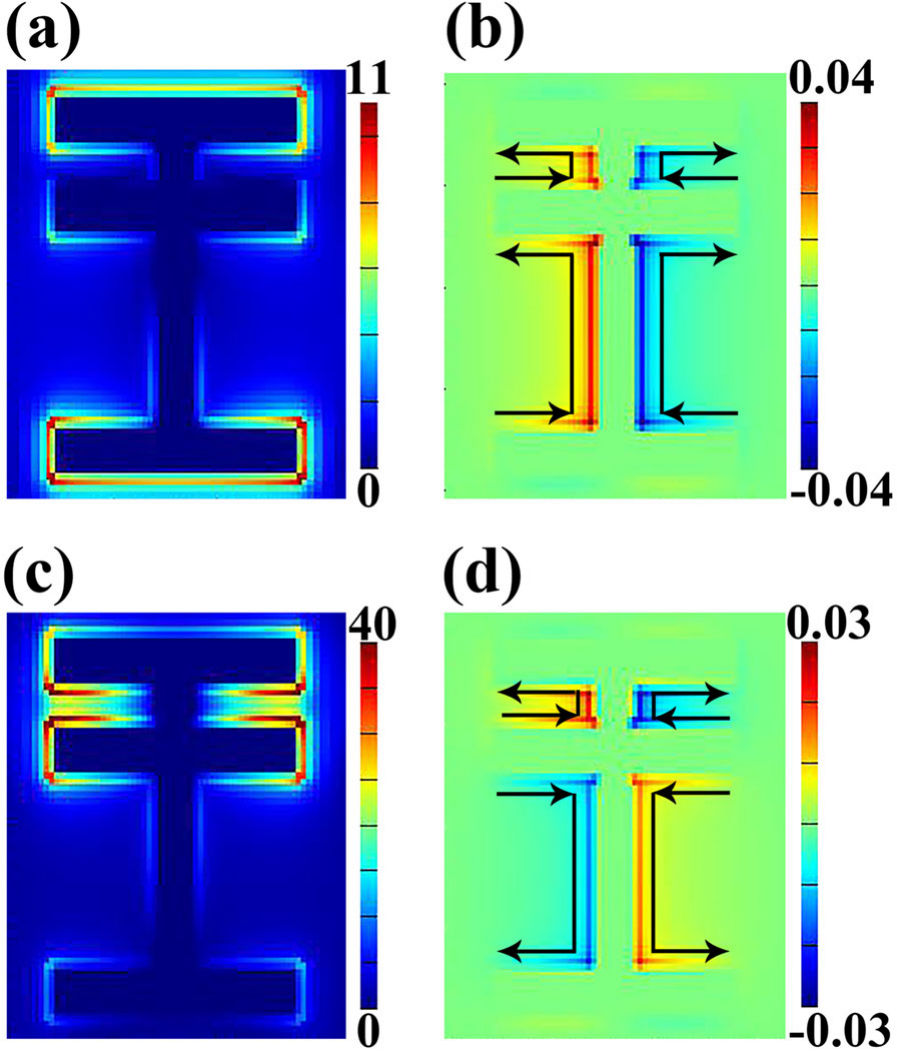
Distributions of electric field ∣E∣ (**a**) and *z* component of magnetic field (H_Z_) (**b**) at 0.430 THz (*R*_s_); distributions of ∣E∣ (**c**) and H_Z_ (**d**) at 0.809 THz (*R*_d_); black arrows in **b** and **d** represent the direction of surface current

In order to figure out the origin of the transmittance curve, the distribution of electric field ∣***E***∣ and the *z* component of magnetic field (*H*_Z_) at the central frequency of two resonant dips are given in Fig. 3. We can find great differences between the field distributions of *R*_s_ and *R*_d_. Figure 3a indicates that the electric field of resonant mode *R*_s_ is mainly concentrated on strip 1 and strip 3, especially the ends of these two strips. However, there is very little electric field distribution on other portions of the structure, including strip 2 and strip 4. Such electric field distribution is owing to the electromagnetic field of the normally incident light whose electric vector ***E*** is along *y*-axis. Therefore, *R*_s_ can be regarded as fundamental resonance (i.e., localized electromagnetic EM (electromagnetic) response) [42]. Besides, the distribution of the *z* component of magnetic field (*H*_Z_) for mode *R*_s_ is shown in Fig. 3b from which we can get surface current distribution. It has been demonstrated that analysis of surface current can serve as a vital method to reveal how the coupling of modes generates Fano resonance [28]. As shown in Fig. 3b, surface current flows from the bottom to the upper part of the structure, contributing to the collection of opposite charges on both sides of strip 1 and strip 3. In contrast, the field distribution at the central frequency of *R*_d_ is rather different. Intense electric field is found around strip 1 and strip 2 (Fig. 3c), which is approximately four times larger than that of the mode *R*_s_. According to the distribution of *H*_Z_ field shown in Fig. 3d, it is clear that the surface current flows upwards between strip 1 and strip 2 while the current between strip 2 and strip 3 flows oppositely. At the macro level, such field distribution can be seen as some kind of charge induction between the horizontal strips. From the perspective of mode coupling, this phenomenon is due to the interaction between the bright mode and dark mode.

Aiming to deepen and quantify our explanation, the spectra of bright mode and dark mode are simulated and the electromagnetic theory of Fano resonance [15, 16] is used in the proposed structure. Figure 2b shows the transmission spectrum of a structure whose periodic unit consists of strips 1, 3, and 4. The resonant mode supported by such a structure can be excited directly by plane wave; therefore, it is “bright mode.” In contrast, a dark mode cannot be excited by a beam of plane wave; it can be excited through a rapidly varying field, for instance, the near field of a dipole [15, 43]. Figure 2c displays the field intensity of the four-strip metamaterial illuminated by a dipole source [44]. Maxwell’s equations form a solid basis of the electromagnetic theory of Fano resonance in nanostructures. According to Maxwell’s equations, electric vector ***E*** obeys the wave equation below:

1 $$ {\in}^{-1}\left(\mathbf{r},\upomega \right)\nabla \times \nabla \times \mathbf{E}\left(\mathbf{r},\upomega \right)-\frac{\upomega^2}{{\mathrm{c}}^2}\mathbf{E}\left(\mathbf{r},\upomega \right)=0 $$

where ω is the frequency of incident beam and ∈(**r**, ω) is the complex dielectric constant of the lossy material. The electric field **E** and the permittivity ∈ are both related to the frequency ω as well as the position vector **r**. Two orthogonal projection operators P and Q can be used to separate wave function ∣**E**> into a bright mode P ∣ **E**> and a dark mode Q ∣ **E**>, i.e., a radiative mode and a nonradiative mode [15, 38]. Through complicated derivation, the ratio of the total field’s strength to the intense of the bright mode can be given as

2 $$ {I}_{\mathrm{a}}\left(\upomega \right)=\frac{{\left(\frac{\upomega^2-{\upomega_{\mathrm{a}}}^2}{2{W}_{\mathrm{a}}{\upomega}_{\mathrm{a}}}+q\right)}^2+b}{{\left(\frac{\upomega^2-{\upomega_{\mathrm{a}}}^2}{2{W}_{\mathrm{a}}{\upomega}_{\mathrm{a}}}\right)}^2+1} $$

where *W*_a_ and ω_a_ are the bandwidth and central frequency of the asymmetric resonance, respectively. The asymmetric parameter *q* and the modulation damping parameter *b* are both indispensible to describe *I*_a_(ω) [15, 16]. The Eq. (2) suggests that *I*_a_(ω) exhibits an asymmetric profile, which finally results in the asymmetric Fano line shape in the transmittance curve.

The intensity of the bright mode *R*_s_ follows a smooth Lorentzian profile. It depends on the frequency ω and obeys the following equation:

3 $$ {I}_{\mathrm{s}}\left(\upomega \right)=\frac{a^2}{{\left(\frac{\omega^2-{\omega_{\mathrm{s}}}^2}{2{W}_{\mathrm{s}}{\omega}_{\mathrm{s}}}\right)}^2+1} $$

of which *W*_s_ and *ω*_s_ are respectively the bandwidth and central frequency of the spectrum displayed in Fig. 2b, and *a* is the maximum value of the resonance’s amplitude. The total strength *I*(*ω*) of the resonance can be calculated by the product of *I*_a_ and *I*_s_, from which we can finally get the transmittance *T*(ω).

4 $$ I\left(\omega \right)={I}_{\mathrm{a}}\left(\omega \right)\times {I}_{\mathrm{s}}\left(\omega \right) $$

5 $$ T\left(\omega \right)=1-I\left(\omega \right) $$

In order to meet the requirement of energy conservation, *a* should not be greater than 1. *W*_a_ and *ω*_a_ can be calculated from the central frequency and bandwidth [15, 16]. The asymmetric parameter *q* as well as the modulation damping parameter *b* can be obtained through the method given by [16]. In this way, we can get the theoretical transmission spectrum of this asymmetric structure. In Fig. 2f, the black curve represents the transmission spectrum given by FDTD method and the red curve gives the results of our calculation based on electromagnetic theory of Fano resonance. The consistent trend of black and red curve indicates that it is reasonable to attribute the resonator’s transmission characteristics to the coupling of the bright mode and dark mode. This conclusion also coincides with the distribution of field in Fig. 3.

The geometric parameter *d* describes the distance between the axis of strip 2 and the central point of the whole structure (Fig. 1c). It can greatly affect the central frequency of transmission dips as well as their transmission coefficients. Corresponding transmission spectra with different *d* are shown in Fig. 2d, e. The black curve and red curve represent the transmission spectrum based on simulation and theoretical calculation, respectively. With *d* changing from 10 to 30 μm, it is clear that a sharp Fano dip deepens, resulting from the increasing coupling strength between the bright mode and dark mode. Furthermore, the central frequency of the mode *R*_d_ exists a distinct blue shift when strip 2 is placed closer to strip 1. Based on LC circuit model, the resonance frequency of *R*_d_ is given by [45].

6 $$ {\omega}_{\mathrm{d}}=\frac{1}{2\uppi \sqrt{\mathrm{LC}/2}}\propto \frac{1}{\mathrm{l}} $$

where *l* is the equivalent length of the corresponding resonator. Equation (6) indicates that the central frequency *ω*_d_ is in inverse proportion to *l*. In our structure, equivalent length *l* is indicated by the length of dot-dash line in Fig. 1c. This is because the field distributions of *R*_d_ are mainly restricted to the strip 1 and 2. The length of strip 1 (and 2) and the distance between the two strips together decide *l.* When *d* increases, the distance between the two strips declines. Hence, as shown in Fig. 1c, the equivalent length decreases when *d* changes from 10 to 30 μm. This leads to the increase of *R*_d_’s resonant frequency.

**Fig. 4:**
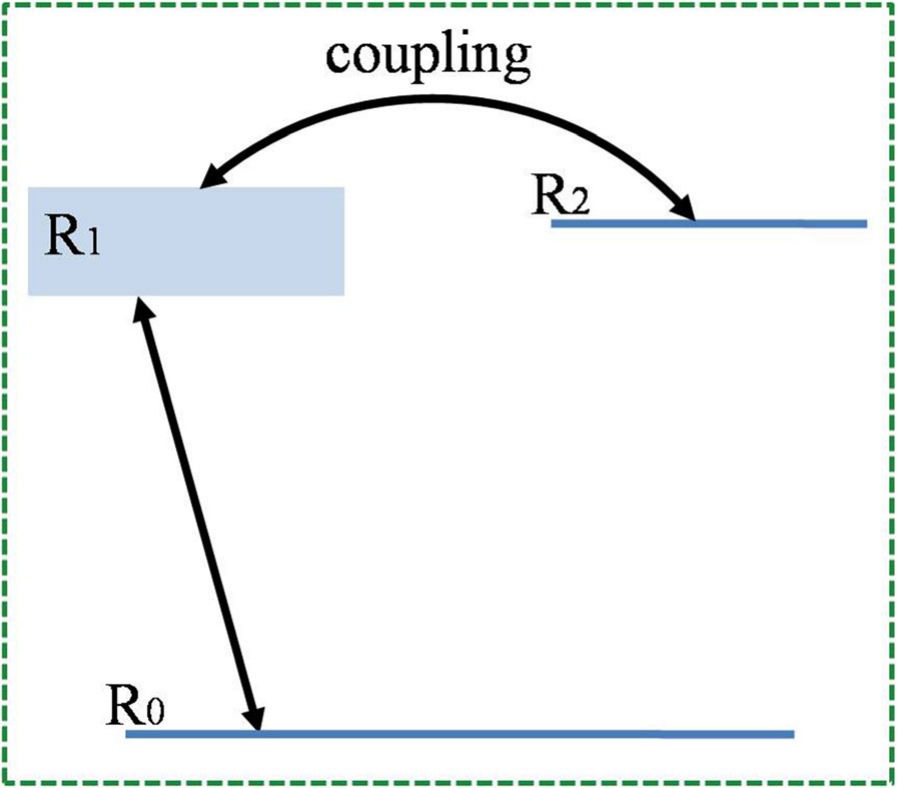
Schematic diagram of the three-level system

According to the theory of Fano resonance suggested by Fano in 1961 [12], the process of autoionization is studied and the asymmetric line shape of the resonance is attributed to the interference between a continuum and a discrete state. This is also the origin of those asymmetric characteristics of the metamaterial resonator presented in this paper. As shown in Fig. 4, a three-level system can be utilized to clarify the transition mechanism of the structure. *R*_0_ serves as the ground state of the whole system. Bright mode *R*_1_ is a radiative mode that can be directly excited by the normally incident beam. In this system, the nonradiative state *R*_2_ can be regarded as a “dark mode” [21] as discussed before. *R*_2_ can be excited through the symmetry breaking. Introducing an asymmetry offers a channel to allow the bright mode to couple with the dark mode, and therefore, leads to the Fano resonance [46].

**Fig. 5:**
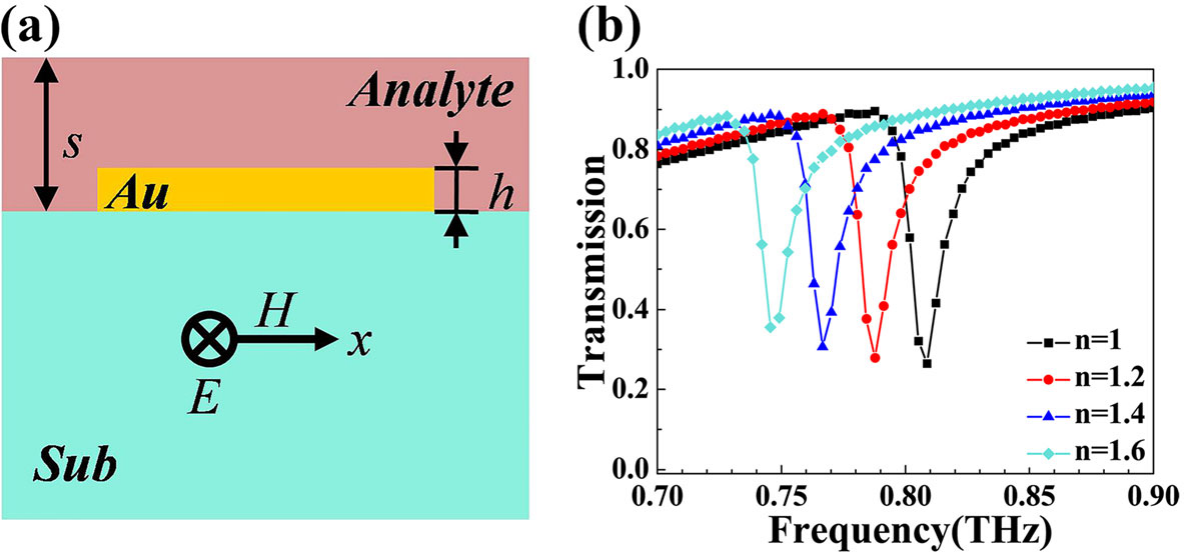
**a** Cross section of the sensing device. **b** Dependence of transmission spectra on the changes of refractive index *n*

The strong interaction between incident electromagnetic wave and the analyte layer makes high-*Q* Fano resonance, a promising method to realize ultra-sensitive detecting of refractive index *n* [8]. The device proposed in Fig. 5a can function as an effective sensor to detect the refractive index *n* of the analyte layer on the top whose thickness is 4 μm. The central frequency of the Fano dip will change with the change of *n*. Therefore, we can get the refractive index by analyzing the resonant frequency of *R*_d_. Figure 5b shows the shift of Fano dip’s resonant frequency in the device. A distinct red shift appears when *n* is increased from 1 to 1.6. The sensing sensitivity *S* is equal to $\frac{\varDelta f}{\varDelta n}$. Here, *S* of the sensor is calculated to be 0.105 THz/RIU (refractive index unit). It is well known that FOM (Figure of merit) is a vital criterion for the performance of a sensor [47]. It can be calculated by FOM = $\frac{S}{\mathrm{linewidth}}$. In this presented structure, the FOM value can reach 7.501, which is on an ideal level [47, 48]. The sensing capability is also usually discussed by FOM* = $\frac{S^{\ast }}{I}$ and S* = $\frac{\varDelta I}{\varDelta n}$, which is related to the detected intensity. The calculation result of S* in this structure is 2.6/RIU. And the FOM* in our structure is calculated to be 10. We also did some work to figure out the variation of the response with the thickness of the analyte layer. Please refer to the Additional file 1.

**Fig. 6:**
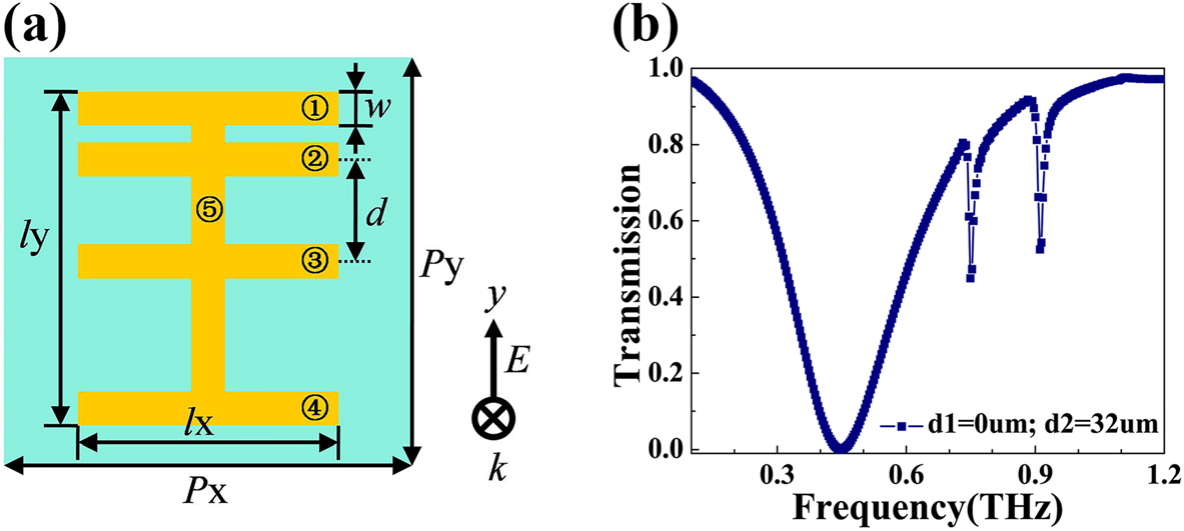
**a** Top view of the proposed five-strip structure. **b** Simulated transmittance curve of the five-strip resonator

Multiple Fano resonances could have applications in many situations. However, most of the plasmonic Fano metamaterials are designed to support single Fano resonance [11, 17]. Therefore, it is not easy for them to realize multiple Fano resonances through the adjustment of structure. In this paper, we realize multiple Fano resonances by adding more horizontal strips into the original design of the metamaterial. We present a five-strip structure as a representative example. The schematic diagram of the five-strip resonator is shown in Fig. 6a. Strips 1, 2, 3, and 4 are in the same size and parallel to each other. Their length is *l*_x_ = 120 μm and width is *w* = 20 μm. Strip 3 is located in the middle, and the distance *d* between the axes of strip 2 and strip 3 is 32 μm. Strip 5 is perpendicular to the other four strips. Its length is *l*_y_ = 150 μm and width is *w* = 20 μm. The boundary conditions and mesh size are kept the same as the simulation of four-strip resonator. The simulation result is shown in Fig. 6b, in which we can clearly find two sharp Fano dips at 0.75 THz and 0.91 THz. The *Q* values of these two dips are respectively 61 and 65. More Fano dips should be generated if more horizontal strips are added into the structure.

## Conclusion

In conclusion, we design a four-strip planar resonator which can support sharp Fano resonance with a high *Q*-value. The bandwidth of the Fano dip is 0.014 THz and its *Q*-factor is 58. The interaction between bright mode and dark mode results in the appearance of the asymmetric Fano profile. The theoretical transmission spectrum is calculated in this paper. Furthermore, multiple high-*Q* Fano resonances can be realized by adding more horizontal strips to the structure. This structure can be applied in sensing and other fields.

## Additional File

Additional file 1: The variation of the response with the thickness of the analyte layer. (DOCX 297 kb)

## Abbreviations

EIT: Electromagnetically induced transparency
EM: Electromagnetic
FOM: Figure of merit
PIT: Plasmon-induced transparency
*Q*: Quality factor
RIU: Refractive index unit
