# Supplementary material for: High-Q Fano Resonance in Terahertz Frequency Based on an Asymmetric Metamaterial Resonator
Source: Nanoscale Res Lett. 2018 Sep 21;13:294. doi: 10.1186/s11671-018-2677-0 (PMC6150870; doi:10.1186/s11671-018-2677-0)
Supplement: Supplementary file 1 — The variation of the response with the thickness of the analyte layer. (DOCX 297 kb) [file 11671_2018_2677_MOESM1_ESM.docx]

**Additional file 1**

**High-Q Fano resonance in terahertz frequency based on an asymmetric metamaterial resonator**

**Qin Xie^1,a^, Guang-Xi Dong^1,b^, Ben-Xin Wang^1,c^, and Wei-Qing Huang^1,d^**

*^1^ School of Science, Jiangnan University, Wuxi 214122, China*

*^a^ Author to whom correspondence should be addressed; electronic mail:* [*1130515207@vip.jiangnan.edu.cn*](mailto:1130515207@vip.jiangnan.edu.cn)

*^b^ electronic mail:* [*1130515112@vip.jiangnan.edu.cn*](mailto:1130515112@vip.jiangnan.edu.cn)

*^c^* *electronic mail:* [*wangbenxin@jiangnan.edu.cn*](mailto:wangbenxin@jiangnan.edu.cn)

*^d^ electronic mail:* [*wqhuangjnu@163.com*](mailto:wqhuangjnu@163.com)

**1. the Variation of the Response with the Thickness of the Analyte Layer**

In order to figure out the variation of response with the thickness of analyte layer, we have simulated the cases of different thickness (2um, 4um, 6um and 8um). Here are the simulating results. Figs.1 (a), (b), (c) and (d) below respectively show the dependence of transmission spectra on the changes of refractive index *n* when thickness is 2um, 4um, 6um and 8um. In all these four cases, we can find a distinct red shift of mode *R*_d_ when the refractive index *n* increases from 1.0 to 1.6. Different analyte thickness may lead to a different degree of frequency shift. With thickness changing from 2um to 8um, such frequency shift becomes more apparent. Aiming to make our analysis more concise, we have calculated S and FOM for all these four cases quantitatively. With thickness being 2um, 4um, 6um and 8um, sensing sensitivity S of the device is 0.064 THz/RIU, 0.105 THz/RIU, 0.129 THz/RIU and 0.146 THz/RIU when FOM of the device is 4.583, 7.501, 9.197 and 10.417, respectively. Figs.2 (a) and (b) below respectively show the dependence of S and FOM on a varying thickness. According to Figs.2 (a) and (b) below, thicker analyte layer will contribute to higher S and FOM, which may increase the effectiveness and sensitivity of refractive index sensing. In addition, based on the tendency shown in Figs.1 (a), (b), (c) and (d) below, we can evidently find that there is a possibility that a thicker or thinner material with a particular refractive index will give the same response as a 4um thick material with a different refractive index.


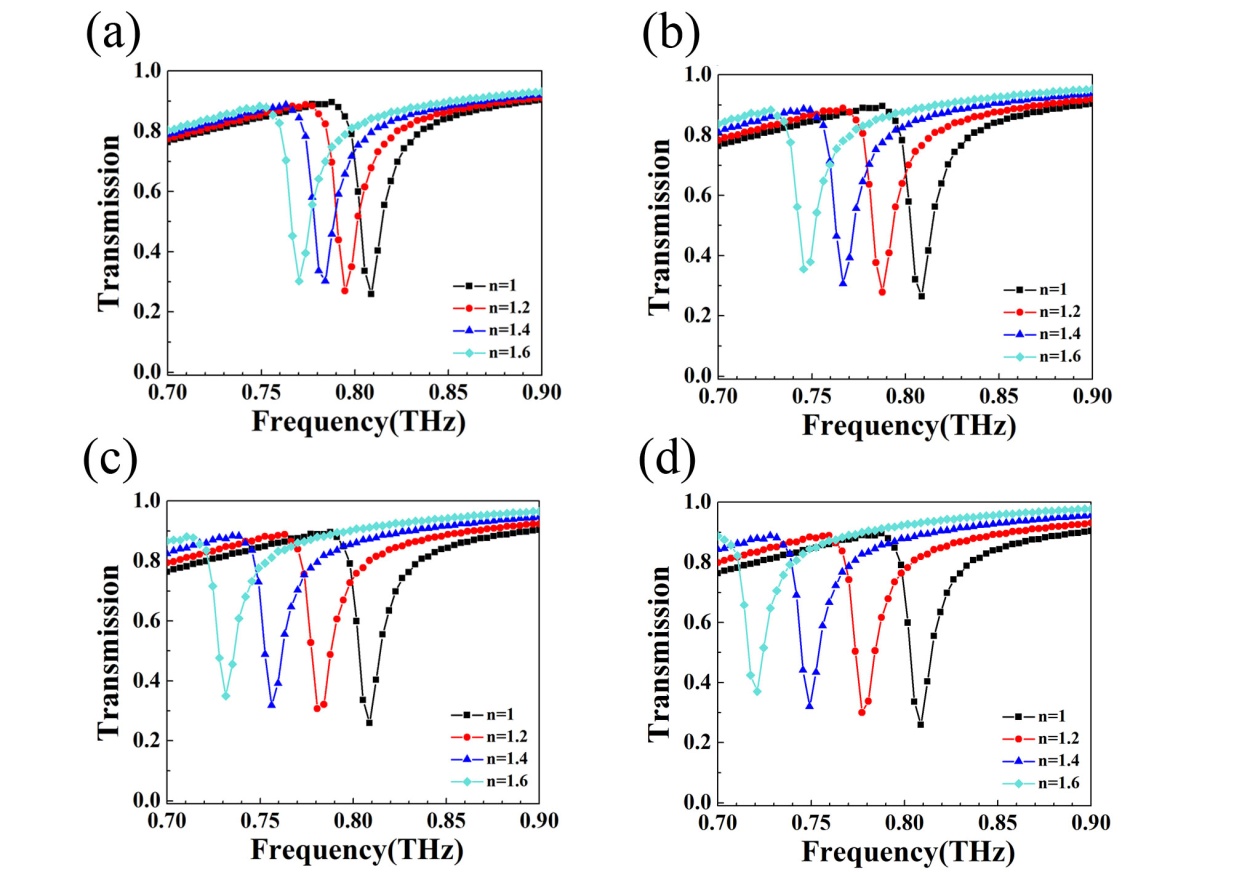


Fig. 1. dependence of transmission spectra on the changes of refractive index *n* when thickness is 2um (**a**), 4um (**b**), 6um (**c**) and 8um (**d**).


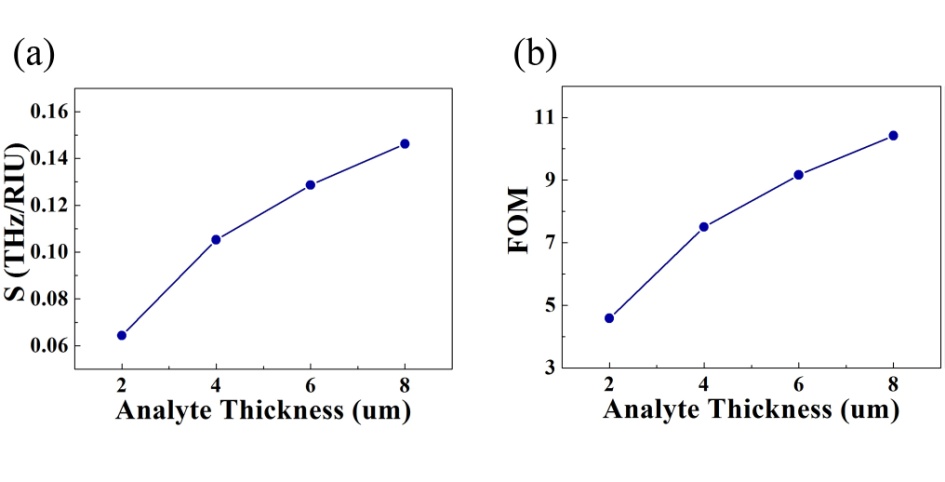


Fig. 2. dependence of S (**a**) and FOM (**b**) on a varying analyte thickness
